# Environmental Contact and Self-contact Patterns of Healthcare Workers: Implications for Infection Prevention and Control

**DOI:** 10.1093/cid/ciz558

**Published:** 2019-09-13

**Authors:** Linh T Phan, Dayana Maita, Donna C Mortiz, Susan C Bleasdale, Rachael M Jones

**Affiliations:** 1 School of Public Health, University of Illinois at Chicago; 2 College of Medicine, University of Illinois at Chicago

**Keywords:** contact transmission, fomites, self-contact, infection control, respiratory infections

## Abstract

**Background:**

Respiratory viruses on fomites can be transferred to sites susceptible to infection via contact by hands or other fomites.

**Methods:**

Care for hospitalized patients with viral respiratory infections was observed in the patient room for 3-hour periods at an acute care academic medical center for over a 2 year period. One trained observer recorded the healthcare activities performed, contacts with fomites, and self-contacts made by healthcare workers (HCWs), while another observer recorded fomite contacts of patients during the encounter using predefined checklists.

**Results:**

The surface contacted by HCWs during the majority of visits was the patient (90%). Environmental surfaces contacted by HCWs frequently during healthcare activities included the tray table (48%), bed surface (41%), bed rail (41%), computer station (37%), and intravenous pole (32%). HCWs touched their own torso and mask in 32% and 29% of the visits, respectively. HCWs’ self-contacts differed significantly among HCW job roles, with providers and respiratory therapists contacting themselves significantly more times than nurses and nurse technicians (*P* < .05). When HCWs performed only 1 care activity, there were significant differences in the number of patient contacts and self-contacts that HCWs made during performance of multiple care activities (*P* < .05).

**Conclusions:**

HCWs regularly contact environmental surfaces, patients, and themselves while providing care to patients with infectious diseases, varying among care activities and HCW job roles. These contacts may facilitate the transmission of infection to HCWs and susceptible patients.

## Teaser

Contact pattern of healthcare workers and patients during care activities for hospitalized patients with viral respiratory infections are described. Healthcare workers regularly contact environmental surfaces that may be contaminated with pathogens and their bodies, putting them at risk for acquiring infection of disseminating pathogens through the contact route.

Environmental surfaces contaminated with pathogens are thought to play an important role in the contact transmission route because pathogens can be transferred to the facial mucous membranes or other sites on a susceptible person via contact by hands or other fomites [1]. Respiratory viruses such as influenza viruses and severe acute respiratory syndrome coronavirus (SARS-CoV) are transmitted, at least in part, through the contact route [2]. These viruses significantly impact the health of healthcare workers (HCWs). During the 2003 SARS-CoV outbreak, healthcare workers accounted for 20% of the cases worldwide [3]. With respect to seasonal influenza, Jones and Xia estimated that tens of thousands of HCWs in acute care settings in the United States are infected through occupational exposure annually [4]. Environmental surfaces in the hospital have been found to be contaminated with influenza viruses and SARS-CoV, including the hospital bed, bedside table, and television remote control [5–7]. Previous work found that patient bodies, bed surfaces, and bedside tables were among the most frequently touched surfaces by HCWs and patients [8, 9]. Thus, contact with these surfaces may put HCWs at risk of infection or of transmitting the pathogens to other susceptible patients and workers.

The objective of this study is to describe the contact patterns of HCWs with environmental surfaces (fomites) and themselves, and the contact patterns of patients with fomites during HCW–patient encounters. All patients were hospitalized with viral respiratory infection in an acute care hospital in an academic medical center. To our knowledge, fomite contact and self-contact patterns of HCWs associated with care activities for patients with viral respiratory infections, all of whom were under transmission-based infection control precautions, have not been studied. This work can inform our understanding of infectious disease transmission pathways, support implementation of quantitative microbial risk assessment, and direct enhanced cleaning activities toward frequently touched surfaces. Furthermore, recognition of environmental contacts and self-contact by HCWs may motivate improved use of personal protective equipment (PPE) and hand hygiene, which we have found to be inadequate among HCWs at this hospital [10].

## METHODS

Care for patients with viral respiratory infections was observed at an acute care hospital (465 beds) from March 2017 to June 2017 and from September 2017 to April 2018. Eligibility, recruitment, and consent of HCWs and patient participants have been described elsewhere [10]. Patients having viral respiratory infections were identified through the hospital MedMined surveillance system. Patients were eligible to participate if they were adults, fluent in English or Spanish, and had a positive respiratory pathogen panel test within 3 days prior to the day of observation. Eligible patient participants were recruited in the patient room and provided written informed consent and authorization to use and disclose health information for the study. This study was approved by the University of Illinois at Chicago Institutional Review Board (protocol number 2015-0990).

Researchers performed observations inside the patient room during a 3-hour period, typically from 8 am to 12 pm. One trained observer recorded contacts with fomites in the environment and self-contacts of the HCW participant while another observer recorded fomite contacts by the patient participant during the encounter using predefined checklists (Supplementary Materials). Observations included only encounters by consented HCWs, and did not include visitor encounters or short interactions between HCWs and patients such as food delivery/tray removal. In addition, we did not observe patient contact with the environment when a HCW was not present or when patients were in the bathroom to respect patient privacy.

If >1 HCW was in the room at the same time, only the contacts of 1 consented HCW were recorded. A contact was defined as any contact made by a gloved or bare hand with a fomite. For example, 1 bedrail contact and 1 patient contact were counted if the HCW touched the patient then moved his/her hand to the bed rail. However, 1 patient contact was counted if the HCW touched the patient multiple times without moving hands to another fomite in between patient contacts.

Herein we focus on (1) fomite contacts by HCWs; (2) fomite contacts by patients; and (3) self-contacts by HCWs. Fomite contacts were summarized based on the proximity to the patient. Fomites in the near-patient zone included bed surface, bed rail, call button, chair, phone, tray table, and bedside table. Fomites in the far-patient zone included light switch, room door, curtain, computer station, trash can, intravenous (IV) pole, IV monitor, isolation stethoscope, sink, toilet, sharps container, vitals machine, blood pressure cuff, medication scanner, blood glucose monitor, thermometer, pulse oximeter, and respiratory supplies. Self-contact locations by HCWs included mask (if used), head area other than mask, torso, hand, lower body, and personal stethoscope (if used). We did not include the personal stethoscope contact in the total self-contact count because not all HCWs used a personal stethoscope during the patient encounters.

We grouped observations by HCW job roles: (1) provider, including attending physicians, resident physicians, nurse practitioners, and medical students (n = 41); (2) nurse, including nurses and nursing students (n = 69); (3) nurse technicians (n = 37); (4) respiratory therapists (n = 9); and (5) others, including physical therapists, environmental service workers, and dieticians (n = 8). Observations were performed across 9 units in the hospital, organized into 4 groups: (1) intensive care units (ICU) including the neurological ICU, medical ICU, and the step-down unit (n = 35); (2) clinical decision unit (CDU) (n = 51); (3) non-ICUs including the rehabilitation/orthopedics unit, general medical-surgical unit, and liver/gastroenterology unit (n = 48); and (4) specialty units including the bone marrow transplant and hematology-oncology units (n = 30).

We have described data management for this study elsewhere [10]. All data analysis was performed with the R Project for Statistical Computing (R Foundation, Vienna, Austria). The maximum likelihood method was used to fit the log-normal, Poisson, and negative binomial distributions to the number of fomite contacts using the function *gofstat* of package “fitdistrplus,” and the best-fit distribution was selected using the Akaike information criterion [11]. Differences of contacts among groups were tested with the Kruskal-Wallis test (KW), followed by pairwise Wilcoxon test (W) to compare 2 groups, with *P* values adjusted using the Bonferroni method. Observations of HCWs were treated as independent because the small number of HCWs who participated repeatedly performed different care activities and the number of replicates was small. To evaluate the potential effect of different observers, total environmental fomite contacts and self-contacts by HCWs were compared between observers using the KW test.

## RESULTS

We observed healthcare activities for 52 patients with viral respiratory infections who were under isolation: 30 were in droplet and contact isolation, 21 were in droplet isolation, and 1 was in contact isolation. Fomite contact and self-contact patterns were observed for 107 HCWs, with 23 HCWs participating more than once for a total of 166 observations. Two HCW observations were excluded from data analysis because we were not able to record their contacts. In addition, we recorded fomite contacts by patients during 155 encounters. No statistically significant difference in the total number of fomite contacts (KW *P* = .21) or self-contacts (KW *P* = .10) by HCWs was identified among the 3 different observers.

In 90% of visits, HCWs contacted the patient with a median of 2 contacts and 11.1 contacts per hour (Table 1). Fomites contacted frequently by HCWs included the tray table (48% of encounters), bed surface (41%), bed rail (41%), computer station (37%), and IV pole (32%) (Table 1). Fomites in the near-patient zone were contacted in 79% of encounters, with a median of 2 contacts and 13.8 contacts per hour. Fomites in the far-patient zone were contacted in 85% of encounters, with a median of 4 contacts and 23.1 contacts per hour (Table 1). The contact rate on fomites in the near-patient zone was significantly lower than the rate in the far-patient zone (W pairwise, *P* < .05). When probability distributions could be fitted to the contact numbers, the negative binomial distribution was the best-fit distribution (Table 1).

**Table 1.** Fomite Contact Patterns of Healthcare Workers (N = 164)

Surface	Median (Min; 75th Percentile; Max) No. of Contacts	No. (%) of Visits With Contacts	Negative Binomial Distribution Parameters^a^	Median (Min; 75th Percentile; Max) Contact Rate, No. per Hour
Patient	2 (0; 4; 12)	148 (90)	n = 3.47; µ = 2.93	11.1 (0; 29.4; 360)
Fomites in near-patient zone				
Tray table	0 (0; 2; 14)	78 (48)	n = 0.56; µ = 1.28	0 (0; 13.7; 180)
Bed surface	0 (0; 1; 12)	67 (41)	n = 0.47; µ = 0.96	0 (0; 5.2; 72)
Bed rail	0 (0; 1; 7)	68 (41)	n = 0.71; µ = 0.83	0 (0; 7.5; 180)
Chair	0 (0; 0; 6)	17 (10)	^b^	0 (0; 0; 15)
Bedside table	0 (0; 0; 5)	13 (8)	^b^	0 (0; 0; 60)
Call button	0 (0; 0; 2)	11 (7)	^b^	0 (0; 0; 7.5)
Phone	0 (0; 0; 1)	1 (1)	^b^	0 (0; 0; 0)
Total	2 (0; 4.3; 32)	66 (79)	n = 1.04; µ = 3.50	13.8 (0; 27.9; 240)
Fomites in far-patient zone				
Computer station	0 (0; 2;10)	61 (37)	n = 0.29; µ = 1.27	0 (0; 13.9; 360)
IV pole	0 (0; 1; 12)	53 (32)	n = 0.25; µ = 0.93	0 (0; 4.6; 60)
Room door	0 (0; 0.3; 3)	41 (25)	^b^	0 (0; 4.8; 180)
Bathroom door	0 (0; 0; 4)	35 (21)	^b^	0 (0; 0; 0; 180)
Sharps container	0 (0; 0; 3)	31 (19)	^b^	0 (0; 0; 30)
Medication scanner	0 (0; 0; 3)	20 (12)	^b^	0 (0; 0; 120)
Vitals machine	0 (0; 0; 7)	28 (17)	^b^	0 (0; 0; 240)
IV monitor	0 (0; 0; 3)	24 (15)	^b^	0 (0; 0; 15)
Isolation stethoscope	0 (0; 0; 3)	24 (15)	^b^	0 (0; 0; 20)
Sink	0 (0; 0; 2)	20 (12)	^b^	0 (0; 0; 60)
Trash can	0 (0; 0; 4)	16 (10)	^b^	0 (0; 0; 24)
Toilet	0 (0; 0; 3)	13 (8)	^b^	0 (0; 0; 120)
Blood pressure meter	0 (0; 0; 2)	13 (8)	^b^	0 (0; 0; 120)
Blood glucose monitor	0 (0; 0; 4)	13 (9)	^b^	0 (0; 0; 30)
Respiratory supplies	0 (0; 0; 5)	8 (5)	^b^	0 (0; 0; 40)
Oxygen device	0 (0; 0; 6)	7 (4)	^b^	0 (0; 0; 40)
Light switch	0 (0; 0; 2)	6 (4)	^b^	0 (0; 0; 9.2)
Curtain	0 (0; 0; 8)	5 (3)	^b^	0 (0; 0; 68.6)
Thermometer	0 (0; 0; 2)	4 (2)	^b^	0 (0; 0; 15)
Total	4 (0; 8.3; 25)	140 (85)	n = 1.23; µ = 5.66	23.1 (0; 45.9; 540)
Fomite grand total	7 (0; 12; 38)	156 (96)	n = 1.83; µ = 9.16	37.8 (0; 95.2; 600)

Abbreviation: IV, intravenous.

^a^Distributions were estimated by the maximum likelihood method.

^b^Fit testing was not statistically significant for Poisson, negative binomial, or log-normal distribution, so no distribution is reported.

HCWs made self-contact in 63% of the encounters, with a median of 1 contact and 5 contacts per hour. The frequency of HCW contacts with specific body parts is summarized in Table 2. The best-fit distributions for the number of self-contacts to the torso, lower body, hands, mask, and the total self-contact were negative binomial (Table 2). Self-contact differed significantly among HCWs with different job roles: Providers and respiratory therapists contacted themselves significantly more times than nurses and nurse technicians (KW, *P* < .05; Table 3).

**Table 2.** Self-contact Patterns of Healthcare Workers (N = 164)

Contact Site	Median (Min; 75th Percentile; Max) No. of Contacts	No. (%) of Visits With Self-contacts	Negative Binomial Distribution Parameters^a^	Median (Min; 75th Percentile; Max) Contact Rate (No. per Hour)
Mask if used	0 (0; 1; 5)	47 (29)	n = 0.44; µ = 0.52	0 (0; 0; 240)
Torso	0 (0; 1; 12)	53 (32)	n = 0.33; µ = 0.76	0 (0; 0; 480)
Hands	0 (0; 1; 7)	44 (27)	n = 0.27; µ = 0.59	0 (0; 5.3; 60)
Lower body	0 (0; 1; 5)	46 (28)	n = 0.35; µ = 0.59	0 (0; 4.6; 120)
Personal stethoscope	0 (0; 0; 4)	20 (12)	^b^	0 (0; 0; 240)
Head^c^	0 (0; 0; 6)	13 (8)	^b^	0 (0; 0; 180)
Total self-contacts^d^	1 (0; 4; 18)	104 (63)	n = 0.63; µ = 2.61	5.0 (0; 18.7; 780)
Test for difference between body part object	***P*** ** < .05**			*P* = .82

^a^Distributions were estimated by the maximum likelihood method.

^b^Fit testing was not statistically significant for Poisson, negative binomial, or log-normal distribution, so no distribution is reported.

^c^Head contacts do not include mask contact.

^d^Total self-contacts do not include personal stethoscope contact.

**Table 3.** Contact Patterns by Job Roles of Healthcare Workers, Hospital Units, and Isolation Types

Characteristic	No. of Observations	Median (Min; 75th Percentile; Max) No. of Contacts				
		Fomites			Self-contacts	Patient Contacts
		Near-patient Zone	Far-patient Zone	Total		
Hospital unit						
ICU	35	2 (0; 4; 16)	4 (0; 6;16)	6 (0; 8.5; 24)	1 (0; 4; 11)	2 (0; 4; 9)
CDU	51	3 (0; 4; 20)	5 (0; 11; 21)	10 (0; 14; 38)	1 (0; 4.5 18)	3 (0; 4; 11)
Non-ICU	48	2 (0; 4; 24)	4 (0; 7; 22)	8.0 (0; 10; 34)	1 (0; 3.3; 13)	2 (0; 3; 10)
Specialty	30	3 (0; 7; 32)	6 (0; 9.8; 25)	8.5 (0; 15.8; 34)	1 (0; 5.8; 11)	2 (0; 6.8; 12)
Test for differences between units		*P* = .24	*P* = .20	***P* < .05**	*P* = .86	*P* = .44
HCW job role						
Provider	41	2 (0; 4; 9)	2 (0; 2; 10)	3 (0; 6; 19)	4 (0; 6; 18)	2 (0; 3; 8)
Nurse	69	3 (0; 5; 24)	5 (0; 9; 25)	9 (0; 15; 34)	1 (0; 2; 11)	2 (0; 4; 12)
Nurse technician	37	2 (0; 4; 32)	7 (0; 10; 21)	10 (0; 14; 34)	1 (0; 2; 10)	3 (0; 4; 7)
Respiratory therapist	9	1 (0; 3; 11)	5 (2; 8; 16)	6 (3; 15; 19)	5 (0; 8; 13)	2 (1; 3; 7)
Others	8	0 (0; 6.3; 20)	8 (0; 12; 18)	8 (0; 15.3; 38)	1 (0; 8; 12)	0 (0; 3.3; 9)
Test for differences between groups		*P* = .17	***P* < .05**	***P* < .05**	***P* < .05**	*P* = .23
Isolation type						
Contact	4	5 (3; 9.8; 24)	8 (2; 9.3; 10)	13 (5; 19; 34)	2 (1; 2.3; 3)	4 (1; 6; 6)
Droplet	61	2 (0; 5; 16)	5 (0; 8; 25)	7 (0; 12; 34)	1 (0; 3; 12)	2 (0; 4; 11)
Contact and droplet	99	3 (0; 4; 32)	4 (0; 9; 22)	7 (0; 12; 38)	2 (0; 5; 18)	2 (0; 4; 12)
Test for differences between groups		*P* = .11	*P* = .44	*P* = .32	*P* = .18	*P* = .77

Abbreviations: CDU, clinical decision unit; HCW, healthcare worker; ICU, intensive care unit.

**Figure 1.**
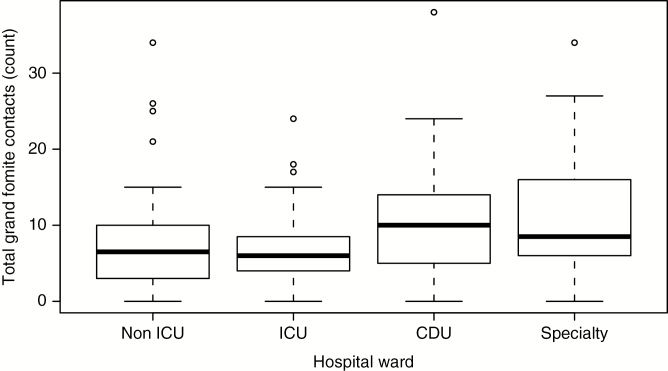
Fomite grand total contact, by hospital ward type. Abbreviations: CDU, clinical decision unit; ICU, intensive care unit.

**Figure 2.**
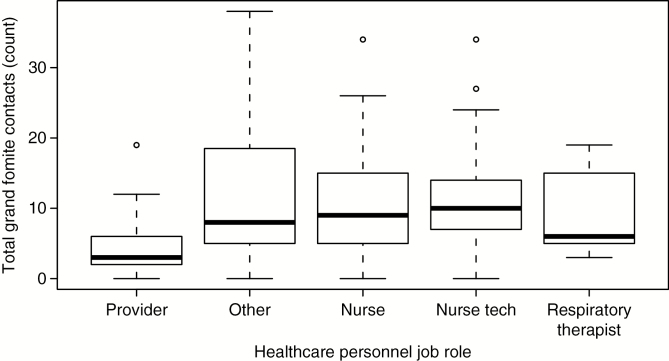
Fomite grand total contact, by healthcare personnel job role.

There were statistically significant differences in the grand total of fomite contacts among hospital units and HCW job roles (Table 3). The median number of fomite contacts in the CDU, non-ICU, and ICU units were 10, 8, and 6, respectively; and were not statistically significantly different in pairwise tests with adjusted *P* values (Figure 1). The median of total fomite contacts made by providers was 3, and was significantly lower than those made by nurses and nurse technicians, with median of 9 and 10 contacts, respectively (W pairwise, *P* < .05; Figure 2). In the far-patient zone, providers made significantly fewer fomite contacts than HCWs in all other job roles (W pairwise, *P* < .05). No differences were observed in the grand total number of fomite contacts by patient isolation category (Table 3).

During 22% of observed encounters, the HCW performed >1 healthcare activity (Table 4). Care activities observed when a single care activity was performed are listed in Table 5, and when HCWs performed >1 care activity, they often performed 1 or more of the activities listed in Table 5. The number of healthcare activities performed was not associated with the duration of the visit, but was positively associated with the total number of fomite contacts in the near-patient and far-patient zones (KW, *P* < .05). The number of fomite contacts when only 1 activity was performed was significantly less than the contacts when 2 or 3 activities happened (W pairwise, *P* < .05). The median visit duration was 13 minutes. When HCWs performed only 1 activity, there were significant differences in the number of patient contacts and self-contacts made during different care activities (KW, *P* < .05) (Table 5). Pairwise comparisons indicated that during cleaning, HCWs had significantly fewer patient contacts than HCWs performing other activities, including vital signs, IV medication, oral medication, and physical examination (W pairwise, *P* < .05). The number of HCW self-contacts made during physical examination was significantly higher than other procedures, including vital signs, IV medication, and oral medication (W pairwise, *P* < .05; Table 5).

**Table 4.** Healthcare Activities and Healthcare Worker Contact Patterns

No. of Activities Performed	No. of Observations	Median (Range) Duration of Visit, min	Median (Min; 75th Percentile; Max) No. of Contacts With Fomites	
			Near-patient Zone	Far-patient Zone
1	128	13 (1; 16)	2 (0; 4; 32)	3 (0; 6; 18)
2	28	13 (1; 17)	3.5 (0; 5.3; 20)	9 (2; 12.3; 21)
3	6	5 (1; 9)	4 (0; 6.3; 9)	16.5 (6; 20.3; 25)
4	2	11 (9; 13)	15.5 (14; 17)	15.5 (9; 18.8; 22)
Total	164	13 (1; 17)	2 (0; 4.3; 32)	4 (0; 8.3; 25)
Test for differences between groups		*P* = .22	***P* < .05**	***P* < .05**

**Table 5.** Healthcare Worker Activities and Contact Patterns When Only 1 Activity Was Performed

Activity	No. of Observations	Median (Min; 75th Percentile; Max) No. of Contacts		
		Fomites	Patient	Self-contact
Physical examination	40	3 (0; 6; 12)	2 (0; 4; 8)	5 (0; 8; 8)
Vital signs	28	9 (0; 12.3; 21)	2.5 (0; 3.3; 6)	1 (0; 2.3; 7)
IV medication	15	7 (4; 12.5; 34)	2 (1; 3; 7)	0 (0; 1; 2)
Oral medication	13	7 (1; 9; 16)	2 (0; 2.1; 3)	1 (0; 2; 4)
Respiratory treatment	8	6 (3; 12.8; 15)	1 (0; 2; 3)	4 (0; 6; 18)
Assisting patient	8	7 (0; 8.5; 25)	2 (0; 3.3; 7)	0.5 (0; 5; 12)
Cleaning	7	9 (4; 13.3; 34)	0 (0; 0; 1)	1 (0; 4; 10)
Verbal interaction	6	1 (0; 2.5; 4.0)	0 (0; 0.8; 2)	0.5 (0; 1; 10)
Other	3	4 (0; 5; 6)	2 (1; 2; 2)	0 (0; 0; 0)
Total	128	6 (0; 9.3; 34)	2 (0; 3; 8)	1 (0; 4; 18)
Test for differences between groups		*P* = .19	***P* < .05**	***P* < .05**

Abbreviation: IV, intravenous.

The probability distributions of patient contacts with fomites could not be described by the negative binomial, Poisson, and log-normal distributions. The most frequently touched fomites by patients were bed surface (92% of the visits), tray table (26%), bed rail (20%), and chair (16%). In 16 of 155 patient observations (10%), the patient was walking in the patient room; otherwise, patients were in bed (Supplementary Materials 1).

## DISCUSSION

Overall, these data indicate that HCWs contact surfaces similarly when providing routine care to patients with viral respiratory infections and other infectious diseases. We found that the patient was the most commonly touched surface, contacted in 91% of the visits, which is consistent with the work of others [8, 9, 12, 13]. Bed surfaces, bed rail, IV pole, computer station, and tray table were among the most highly touched fomites by both HCWs and patients in this study, which is consistent with the work of others [8, 9]. The median contact rate on all fomites by HCWs was 37.8 contacts per hour, which is lower than the 93.1 contacts per hour reported by Cheng et al [9]. As many of these surfaces have been found to be contaminated with respiratory viruses and multidrug-resistant bacteria [5–7, 14], transmission via environmental surfaces is very plausible. The number of contacts by HCWs with patients and fomites and the performance of respiratory care activities have been associated with bacterial contamination on the gloves and hands of HCWs after patient care [15, 16]. This should motivate HCWs to reduced fomite contacts, improved cleaning and disinfection, and hand hygiene to reduce the potential for disease transmission.

The patient encounter duration in our study lasted from 1 to 17 minutes, with a median of 13 minutes. The time range is narrower than has been previously reported, which included a range of 0.5–38 minutes (median, 2 minutes) and a range of 1–124 minutes (median, 3 minutes) [9, 17]. The difference in range may be due to the nature of care activities, which were not described in the previous studies. Furthermore, the higher median in this study may be due to the fact that we intentionally did not observe HCW-patient encounters anticipated to be very short and to not involve contacts with the patient or fomites. In our study, HCWs performed >1 healthcare activity in 22% of the observations, but the number of activities was not associated with the duration of the visit. The combinations of activities performed, however, did not necessarily coincide with defined roles of HCWs.

Our results showed that HCWs touched a variety of portable medical devices that can be used with multiple patients such as the computer station, vitals machine, blood pressure cuff, and blood glucose monitor, and that these devices were used by HCWs with different job roles (Table 3). These results were consistent with the findings of Smith et al [13], in which blood pressure stands and computer stations were handled in patient rooms during 13% and 26% of the HCW visits, respectively. Computer stations in the hospital we studied are permanently present in the patient room of the ICU and CDU wards, but could be portable (on wheels) in the other wards. A simulation study in a long-term care facility using a DNA marker showed that pathogens could disseminate from a television remote control to the hands of patients, to the room surfaces, and to the vital signs machine and medication cart [18]. Therefore, portable medical devices could transmit pathogens within and between patient rooms if they are not properly cleaned. The Centers for Disease Control and Prevention has recommended that “noncritical” surfaces should be disinfected after use on each patient at the minimum [19]. At the hospital in which this study was conducted, the policy is that reusable medical equipment is disinfected between patients, but cleaning behaviors and efficacy was not observed in this study. Jinadatha et al [8] observed that portable medical devices were cleaned 17 times over 274 observations, though it is not clear if this cleaning is sufficient or insufficient. Cleaning of portable medical devices should be further explored as an infection prevention strategy.

Self-contact could transfer infectious pathogens from HCWs’ gloved or bare hands to their PPE, clothing, and skin. To our knowledge, self-contact patterns of HCWs during patient encounters have not been previously explored. In our study, providers and respiratory therapists, who often performed a physical examination and respiratory treatment, had a significantly higher number of self-contacts than HCWs with other roles. The mask and torso were the body parts most commonly touched by HCWs. Bacterial pathogens, including multidrug-resistant organisms, have been found on the gloves, gown, scrubs, and hands of HCWs working in ICUs [20, 21]. Brady et al [22] found that, upon contact during doffing, filtering face piece respirators contaminated with bacteriophage and fluorescein via droplets and droplet nuclei transferred materials to hands; a similar effect can be expected for masks. Doffing of PPE ensembles used for contact-transmissible diseases has been found to result in contamination of HCWs’ hands and, less commonly, faces with surrogate materials placed on the PPE to simulate contamination by pathogen-containing body fluids [23–25]. Our observations of self-contact by HCWs highlight the potential for pathogen transfer between clothing, PPE, and hands.

Mathematical modeling and quantitative microbial risk assessment can help to interpret these contact data for infectious disease transmission. Mechanistic mathematical modeling describes how pathogens move through the environment, to estimate the magnitude of exposure among HCWs and patients, while quantitative microbial risk assessment estimates the probability of infection given an exposure in a susceptible person. Previous applications of this approach have characterized the contribution of different transmission routes to influenza risk [26] and the burden of occupationally acquired influenza in acute care hospitals [4]. Data collected about contact patterns in this study can inform the design and parameter values in such mechanistic exposure models, and will be explored in future work in conjunction with measured pathogen values.

This study has several limitations. First, patient contact patterns were not fully characterized because we did not record patient contacts when a HCW was not present or when patients were in the bathroom. While we did observe that 10% of patients were walking during the HCW encounter, patients observed in bed may or may not have been mobile at other times, and we did not consider patient mobility in this analysis. Second, our findings did not take into account the activities of visitors, who have been found to touch many of the same environmental surfaces touched by HCWs and patients [9]. Third, there are some items in the far patient zone that may contact the patient or move into the near-patient zone when not observed (ie, stethoscope, pulse oximetry). Finally, this is an observational study, so it may be subject to the Hawthorne effect, in which participants change their behaviors because they know they are being observed. The impact of the Hawthorne effect, however, is likely to be small as HCWs continued to provide care as needed by patients, and during this study we observed poor compliance with the use and doffing of PPE [10].

Overall, the data obtained in this study suggest that HCWs are at risk of acquiring or transmitting contact-transmissible pathogens because they touch infected patients and multiple fomites that may be contaminated with respiratory viruses during patient care activities.

## Supplementary Data

Supplementary materials are available at *Clinical Infectious Diseases* online. Consisting of data provided by the authors to benefit the reader, the posted materials are not copyedited and are the sole responsibility of the authors, so questions or comments should be addressed to the corresponding author.

ciz558_suppl_Supplementary_Information

## Notes

***Acknowledgments.*** The authors thank Charissa Fritzen-Pedicini, Rachel Weber, and Maryshe Zietman for their helpful assistance in data collection. Data will be available from the corresponding author or from the University of Illinois at Chicago Intellectual Property in Digital form available in an Open environment (hhtps://indigo.uic.edu/) within 6 months of publication.

***Financial support.*** This work was supported by the CDC (cooperative agreement 1U54CK000445-01).

***Supplement sponsorship.*** This article appears as part of the supplement “Personal Protective Equipment for Preventing Contact Transmission of Pathogens: Innovations from CDC’s Prevention Epicenters Program,” sponsored by the CDC’s Prevention Epicenters Program.

***Potential conflicts of interest.*** All authors report no potential conflicts. All authors have submitted the ICMJE Form for Disclosure of Potential Conflicts of Interest. Conflicts that the editors consider relevant to the content of the manuscript have been disclosed

## References

[1] Siegel JD, Rhinehart E, Jackson M, Chiarello L; Centers for Disease Control and Prevention. 2007 guideline for isolation precautions: preventing transmission of infectious agents in healthcare settings Available at: http://www.cdc.gov/hicpac/pdf/isolation/Isolation2007.pdf. Accessed 13 November 2016. 10.1016/j.ajic.2007.10.007 PMC7119119 18068815

[2] Kutter JS, Spronken MI, Fraaij PL, Fouchier RA, Herfst S Transmission routes of respiratory viruses among humans. Curr Opin Virol 2018; 28:142–51. 29452994 10.1016/j.coviro.2018.01.001 PMC7102683

[3] Sepkowitz KA, Eisenberg L Occupational deaths among healthcare workers. Emerg Infect Dis 2005; 11:1003–8. 16022771 10.3201/eid1107.041038 PMC3371777

[4] Jones RM, Xia Y Annual burden of occupationally-acquired influenza infections in hospitals and emergency departments in the United States. Risk Anal 2018; 38:442–53. 28697286 10.1111/risa.12854

[5] Dowell SF, Simmerman JM, Erdman DD, et al. Severe acute respiratory syndrome coronavirus on hospital surfaces. Clin Infect Dis 2004; 39:652–7. 15356778 10.1086/422652 PMC7107915

[6] Killingley B, Greatorex J, Digard P, et al. The environmental deposition of influenza virus from patients infected with influenza A(H1N1)pdm09: implications for infection prevention and control. J Infect Public Health 2016; 9:278–88. 26653976 10.1016/j.jiph.2015.10.009

[7] Booth TF, Kournikakis B, Bastien N, et al. Detection of airborne severe acute respiratory syndrome (SARS) coronavirus and environmental contamination in SARS outbreak units. J Infect Dis 2005; 191:1472–7. 15809906 10.1086/429634 PMC7202477

[8] Jinadatha C, Villamaria FC, Coppin JD, et al Interaction of healthcare worker hands and portable medical equipment: a sequence analysis to show potential transmission opportunities. BMC Infect Dis 2017; 17:800. 29281998 10.1186/s12879-017-2895-6 PMC5745722

[9] Cheng VC, Chau PH, Lee WM, et al. Hand-touch contact assessment of high-touch and mutual-touch surfaces among healthcare workers, patients, and visitors. J Hosp Infect 2015; 90:220–5. 25929790 10.1016/j.jhin.2014.12.024

[10] Phan LT, Maita DC, Mortiz DC, et al Personal protective equipment doffing practices of healthcare personnel. J Occup Environ Hyg 2019. doi:10.1080/15459624. 2019.1628350 10.1080/15459624.2019.1628350 PMC7157959 31291152

[11] Delignette-Muller ML fitdistrplus: an R package for fitting distributions 2014 Available at: http://r-forge. Accessed 31 July 2018.

[12] Raboud J, Saskin R, Wong K, et al. Patterns of handwashing behavior and visits to patients on a general medical ward of healthcare workers. Infect Control Hosp Epidemiol 2004; 25:198–202. 15061409 10.1086/502377

[13] Smith SJ, Young V, Robertson C, Dancer SJ Where do hands go? An audit of sequential hand-touch events on a hospital ward. J Hosp Infect 2012; 80:206–11. 22297169 10.1016/j.jhin.2011.12.007

[14] Gavaldà L, Pequeño S, Soriano A, Dominguez MA Environmental contamination by multidrug-resistant microorganisms after daily cleaning. Am J Infect Control 2015; 43:776–8. 25907783 10.1016/j.ajic.2015.03.009

[15] Hayden MK, Blom DW, Lyle EA, Moore CG, Weinstein RA Risk of hand or glove contamination after contact with patients colonized with vancomycin-resistant *Enterococcus* or the colonized patients’ environment. Infect Control Hosp Epidemiol 2008; 29:149–54. 18179370 10.1086/524331

[16] Pessoa-Silva CL, Dharan S, Hugonnet S, et al. Dynamics of bacterial hand contamination during routine neonatal care. Infect Control Hosp Epidemiol 2004; 25:192–7. 15061408 10.1086/502376

[17] Cohen B, Hyman S, Rosenberg L, Larson E Frequency of patient contact with health care personnel and visitors: implications for infection prevention. Jt Comm J Qual Patient Saf 2012; 38:560–5. 23240264 10.1016/s1553-7250(12)38073-2 PMC3531228

[18] Alhmidi H, Koganti S, Cadnum JL, Jencson AL, John A, Donskey CJ Dissemination of a nonpathogenic viral DNA surrogate marker from high-touch surfaces in rooms of long-term care facility residents. Am J Infect Control 2017; 45:1165–7. 28526313 10.1016/j.ajic.2017.04.007

[19] Centers for Disease Control and Prevention. Infection control 2016 Available at: https://www.cdc.gov/infectioncontrol/guidelines/disinfection/index.html. Accessed 15 October 2018.

[20] Munoz-Price LS, Arheart KL, Mills JP, et al Associations between bacterial contamination of health care workers’ hands and contamination of white coats and scrubs. Am J Infect Control 2012; 40:e245–8. 22998784 10.1016/j.ajic.2012.03.032

[21] Morgan DJ, Liang SY, Smith CL, et al. Frequent multidrug-resistant *Acinetobacter baumannii* contamination of gloves, gowns, and hands of healthcare workers. Infect Control Hosp Epidemiol 2010; 31:716–21. 20486855 10.1086/653201 PMC3010849

[22] Brady TM, Strauch AL, Almaguer CM, et al. Transfer of bacteriophage MS2 and fluorescein from N95 filtering facepiece respirators to hands: measuring fomite potential. J Occup Environ Hyg 2017; 14:898–906. 28650715 10.1080/15459624.2017.1346799 PMC5705010

[23] Casanova L, Alfano-Sobsey E, Rutala WA, Weber DJ, Sobsey M Virus transfer from personal protective equipment to healthcare employees’ skin and clothing. Emerg Infect Dis 2008; 14:1291–3. 18680659 10.3201/eid1408.080085 PMC2600382

[24] Casanova LM, Rutala WA, Weber DJ, Sobsey MD Effect of single- versus double-gloving on virus transfer to health care workers’ skin and clothing during removal of personal protective equipment. Am J Infect Control 2012; 40:369–74. 21831480 10.1016/j.ajic.2011.04.324 PMC7115263

[25] Kwon JH, Burnham C-AD, Reske KA, et al Assessment of healthcare worker protocol deviations and self-contamination during personal protective equipment donning and doffing. Infect Control Hosp Epidemiol 2017; 38:1077–83. 28606192 10.1017/ice.2017.121 PMC6263164

[26] Nicas M, Jones RM Relative contributions of four exposure pathways to influenza infection risk. Risk Anal 2009; 29:1292–303. 19558389 10.1111/j.1539-6924.2009.01253.x

