# Supplementary material for: Environmental Contact and Self-contact Patterns of Healthcare Workers: Implications for Infection Prevention and Control
Source: Clin Infect Dis. 2019 Sep 13;69(Suppl 3):S178–84. doi: 10.1093/cid/ciz558 (PMC6761362; doi:10.1093/cid/ciz558)
Supplement: ciz558_suppl_Supplementary_Information [file ciz558_suppl_supplementary_information.docx]

Environmental contact and self-contact patterns of healthcare workers: Implications for infection prevention and control

**Supplemental Online Materials**

Linh T. Phan, PhD^a^

Dayana Maita, MD^b^

Donna C. Mortiz, MD^b^

Susan C. Bleasdale, MD^b^

Rachael M. Jones, PhD, CIH^a^*

for the CDC Prevention Epicenters Program

^a^ School of Public Health, University of Illinois at Chicago, Chicago, IL, USA

^b^ College of Medicine, University of Illinois at Chicago, Chicago, IL, USA

^*^ Corresponding Author: Rachael M. Jones, School of Medicine, University of Utah, 391 Chipeta Way Suite C, Salt Lake City, UT 84108, 801-585-0451 (phone), 801-581-7224 (fax)

Supplemental Online Material 1: Fomite contact patterns of patients (N= 156)

| **Fomite Contacted** | **Median**  **(Min, 75^th^, Max)** | **No. (%) with contact** | **Negative binomial distribution parameter*** |
| --- | --- | --- | --- |
| *Near patient zone* | | |  |
| Bed surface | 1 (0; 1; 2) | 143 (92%) | ** |
| Bed rail | 0 (0; 0; 2) | 25 (20%) | ** |
| Call button | 0 (0; 0; 2) | 8 (10%) | ** |
| Chair | 0 (0; 0; 3) | 27 (16%) | ** |
| Phone | 0 (0; 0; 1) | 16 (10%) | ** |
| Tray table | 0 (0; 0; 5) | 34 (26%) | ** |
| Bedside table | 0 (0; 0; 1) | 3 (7%) | ** |
| **Total** | **1 (1; 2; 7)** | **0 (0%)** | ****** |
| *Far patient zone* | | |  |
| Toilet | 0 (0; 0; 1) | 22 (14%) | ** |
| Bathroom door | 0 (0; 0; 3) | 15 (10%) | ** |
| IV pole | 0 (0; 0; 2) | 9 (6%) | ** |
| Light switch | 0 (0; 0; 0) | 0 (0%) | ** |
| Room door | 0 (0; 0; 0) | 0 (0%) | ** |
| Computer station | 0 (0; 0;0) | 0 (0%) | ** |
| Sink | 0 (0; 0; 0) | 0 (0%) | ** |
| IV monitor | 0 (0; 0; 0) | 0 (0%) | ** |
| **Total** | **0 (0; 0; 4)** | **16 (10%)** | ****** |
| **Grand Total** | **1 (1; 2; 8)** | **0 (0%)** | ****** |

*** Fit testing was not statistically significant for poison, negative binomial, or lognormal distribution so no distribution is reported*

Supplemental Online Material 2: Healthcare Worker Observation Form

| **HCW Record of Contact Frequency Form** | | | | **Observation Form** | | | |
| --- | --- | --- | --- | --- | --- | --- | --- |
| **Experiment Code** | | | | **HCW Record of Activities** | | | |
| **HCW code** | | **Observation Code** | **Date** | **HCW type** | | | |
| Mark with a “\|” for every contact | | | | Physical exam |  | BIPAP/CPAP |  |
| HCW Contact with themselves | | HCW hand contact with the environment and patient | | Blood draw |  | Nasal swabbing |  |
| 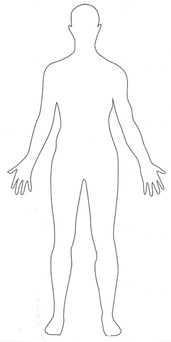 |  |  | | IV Medication |  | Suctioning |  |
|  | |  |  | IV line placement |  | Intubating |  |
|  |  |  |  | Dialysis |  | Bagging |  |
|  |  |  |  | Helping patient to bathroom |  | Respiratory treatment |  |
|  |  |  |  | Room cleaning |  | Dressing change/location |  |
|  |  |  |  | Flushing toilet |  | PT/OT |  |
|  |  |  |  | Bed making |  | Oral medication |  |
|  |  |  |  | Vital signs |  | Resuscitation |  |
|  |  |  |  | Other |  |  |  |

Supplemental Online Material 3: Patient Observation Form

| **Patient Contact Frequency Observation Form** | | | | | | |
| --- | --- | --- | --- | --- | --- | --- |
| **Experiment Code** | | **Date** | | | | |
| **Observation Code** | | | | | | |
| Mark with a \| for every contact in the order it occurred  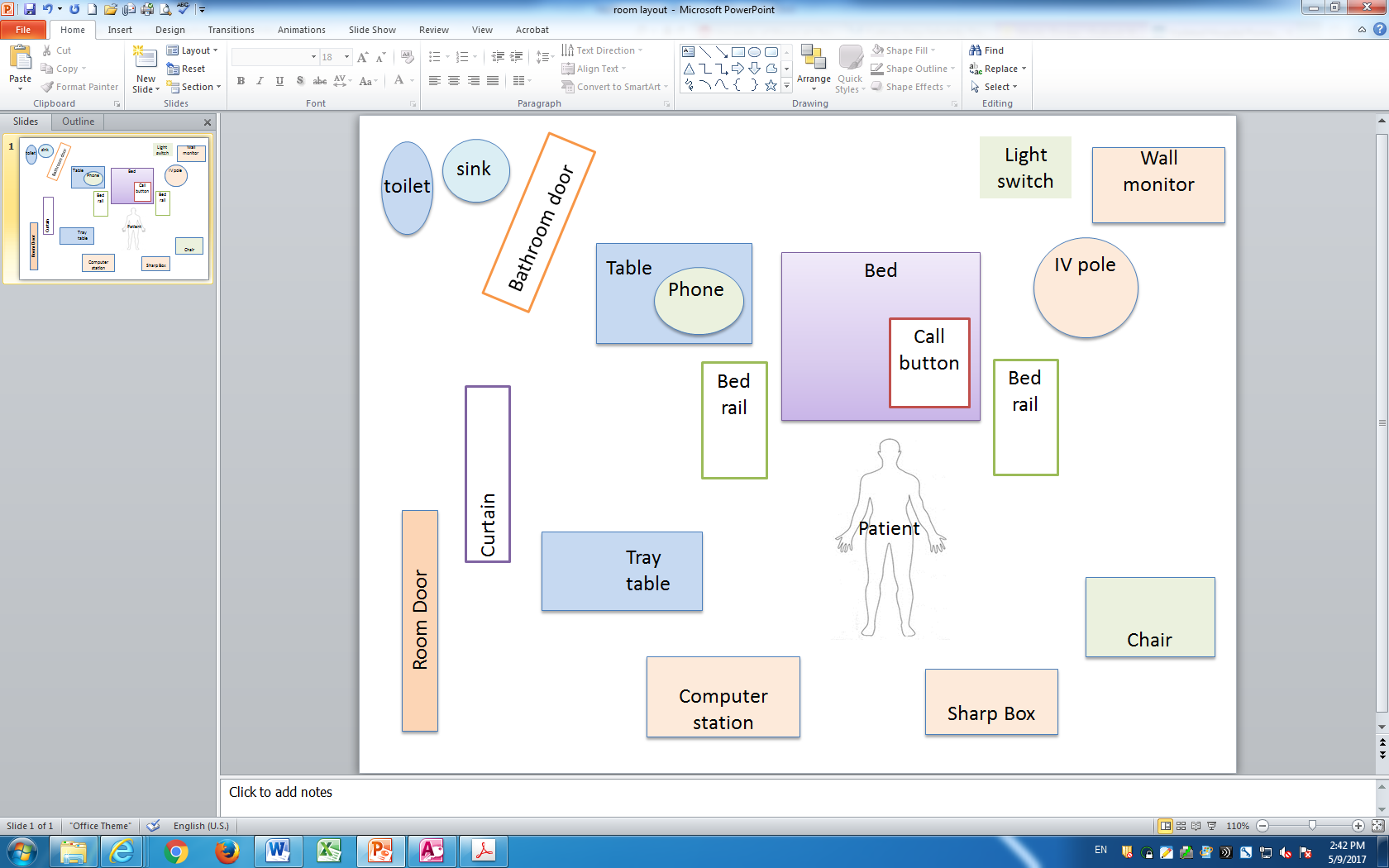 | | | | | | |
| **Clinical appearance/Symptoms and signs** | | | | | | |
| **Respiratory** | **Check if yes** | | **GI** | | | **Check if yes** |
| Coughing/# of times |  | | Vomiting/# of times | | |  |
| Sneezing/# of times |  | | Diarrhea/# of times | | |  |
| Blowing nose/ # of times |  | | Walking | | |  |
| In bed |  | | Other | | |  |
| **Checklist** | | | | **Check if Yes** | **Initial** | |
| **Activity and clinical appearance observed and marked** | | | |  |  | |
